# Statistical inference and effect measures in abstracts of randomized controlled trials, 1975–2021. A systematic review

**DOI:** 10.1007/s10654-023-01047-8

**Published:** 2023-09-16

**Authors:** Andreas Stang, Kenneth J Rothman

**Affiliations:** 1 Institute for Medical Informatics, Biometry, and Epidemiology, University of Duisburg-Essen, University Hospital of Essen, Hufelandstr. 55, 45147 Essen, Germany; 2 School of Public Health, Department of Epidemiology, Boston University, 715 Albany Street, Boston, MA 02118 USA

**Keywords:** Statistics, Confidence intervals, Statistics and numerical data, Randomized controlled trials

## Abstract

**Objective:**

To examine the time trend of statistical inference, statistical reporting style of results, and effect measures from the abstracts of randomized controlled trials (RCTs).

**Study desgin and settings:**

We downloaded 385,867 PubMed abstracts of RCTs from 1975 to 2021. We used text-mining to detect reporting of statistical inference (p-values, confidence intervals, significance terminology), statistical reporting style of results, and effect measures for binary outcomes, including time-to-event measures. We validated the text mining algorithms by random samples of abstracts.

**Results:**

A total of 320 676 abstracts contained statistical inference. The percentage of abstracts including statistical inference increased from 65% (1975) to 87% (2006) and then decreased slightly. From 1975 to 1990, the sole reporting of language regarding statistical significance was predominant. Since 1990, reporting of p-values without confidence intervals has been the most common reporting style. Reporting of confidence intervals increased from 0.5% (1975) to 29% (2021). The two most common effect measures for binary outcomes were hazard ratios and odds ratios. Number needed to treat and number needed to harm are reported in less than 5% of abstracts with binary endpoints.

**Conclusions:**

Reporting of statistical inference in abstracts of RCTs has increased over time. Increasingly, p-values and confidence intervals are reported rather than just mentioning the presence of “statistical significance”. The reporting of odds ratios comes with the liability that the untrained reader will interpret them as risk ratios, which is often not justified, especially in RCTs.

**Supplementary Information:**

The online version contains supplementary material available at 10.1007/s10654-023-01047-8.

## Introduction

The most informative way to report findings from randomized controlled trials (RCTs) is to provide an estimate of effect measure, along with a quantified description of its precision [1]. Among effect measures, absolute measures of effect (rate or risk difference) are preferred over relative, or ratio measures, of effect (rate ratio, risk ratio, hazard ratio). The odds ratio converts risks to risk-odds before taking the ratio, and is therefore more difficult to interpret. Incidence odds ratios and risk ratios are close in value when risks are either small or close together, but begin to differ appreciably as the risks depart from each other and at least one of them surpasses 10%. The number needed to treat (NNT) is the inverse of the absolute risk reduction; it suffers from statistically unpleasant properties [2].

Commonly used measures of precision include the confidence interval and the standard error (e.g [3].). Reporting significance language, numerical p-values, or thresholds for p-values, in addition to the effect-measure estimate, does not provide direct information on the precision of the effect-measure estimate, although confidence intervals can be approximately inferred from a point estimate and a numerical p-value, if the numerical p-value is reported with sufficient precision. From this perspective, we suggest a hierarchy from the most informative to the least informative reporting of statistical inference in the context of effect-measures estimates [1]: (1) an estimate that includes confidence intervals; (2) a point estimate combined with a p-values, either as a numerical quantity (e.g. p = 0.03) or as a threshold (p ≤ 0.05), without an accompanying confidence interval; and (3) a qualitative expression of significance (significant or not significant) without an accompanying p-value or confidence interval.

The CONSORT (Consolidated Standards of Reporting Trials) statement for the reporting of RCTs was developed in the mid 1990s [4, 5]. CONSORT states that authors should report a measure of precision, such as a confidence interval, in addition to an estimate of the effect measure. Furthermore, since 2010, it recommends the presentation of both absolute and relative effect-measures estimates for binary outcomes [6]. CONSORT does not specify whether these measures should be reported in the abstract as well as the full text.

Many journals have adopted CONSORT as a requirement. Our objective was to use PubMed to investigate how much, if any, improvement has occurred in the reporting style of effect-measure estimates and statistical inference in the abstracts of RCTs from 1975 to 2021.

## Material & methods

We searched PubMed (https://www.ncbi.nlm.nih.gov/pubmed, accessed August 17, 2022) for abstracts about RCTs by use of an elaborated search strategy refined by a medical librarian. The search included search fields such as publication type, language, publication date, title and abstract searches. We restricted the search to abstracts published in English between 1975 and 2021 and excluded phase II and phase III RCTs (Supplementary File).

We used a previously developed and validated rule-based text mining algorithm programmed in SAS 9.4 (SAS Institute, Cary, North Carolina) to identify the presence of confidence intervals, numerical p-values (e.g. p = 0.03) or comparisons of p-values with thresholds (e.g., p < 0.01), and language describing statistical significance [7–10]. The classification into the reporting style of statistical inference was made independently of the presentation of effect measures related to dichotomous outcomes.

Using another text mining algorithm, we identified the reporting of effect-measure estimates related to dichotomous outcomes (e.g. death, progression, relapse, etc.) analyzed as person-count data or person-time data. Included effect measures were Risk Difference (RD), Rate Difference, Risk Ratio (RR), Rate Ratio, Hazard Ratio, Odds Ratio (OR), Number Needed to Treat (NNT) and Number Needed to Harm (NNH). The algorithm is unable to distinguish between the different varieties of ORs (prevalence or incidence ORs).

We drew several time-stratified random samples to validate the results of the search algorithms. We compared manually the results of the algorithm with the original text of the abstracts. The validation work was done by the first author. For the four characteristics related to statistical inference, i.e. p threshold, numerical p-value, significance language, and confidence interval reporting, we detected a total of 18 errors out of 180 abstracts (18/(4 × 180) = 2.5%) (Supplementary Table 1). Eleven of the 18 errors arose from the well-known ambiguity of authors using the term “significance” to refer to clinical significance as opposed to statistical significance [7, 10]. The combined sensitivity and specificity of the algorithm were 98% (95%CI 96–99) and 97% (95%CI 95–98), respectively (Supplementary Table 2).

For effect-measure estimates of interest, we drew time-stratified random samples of abstracts for which the algorithm predicted the presence of an effect-measure estimate of interest. We also drew a time-stratified random sample of abstracts where the algorithm predicted the absence of an effect-measure estimate of interest, but a keyword (for relative effect measures: “ratio”, for difference measures of effect: “difference”) was present. We then manually checked the correctness of the algorithm. With the exception of the algorithm for NNH, the algorithm worked without errors. The algorithm for NNH produced one false-positive in a random sample of 20 abstracts.

### Statistical analysis

Based on the four characteristics per abstract, we were able to categorize abstracts containing any statistical inference as follows: reporting of confidence intervals, reporting of p-values, either as numerical values or as thresholds, and reporting of significance language. In addition, we also hierarchically categorized every abstract with statistical inference into one of three mutually exclusive categories: (1) reporting of confidence intervals, (2) reporting of p-values, either as numerical values or as thresholds, without confidence intervals, and (3) significance language only without p-values and without confidence intervals. We calculated the proportions of the reporting styles by calendar year, by calendar period (1975–1979, 1980–1984, …, 2010–2014, 2015–2021) and overall.

For the subvalidation study that assessed the sensitivity and specificity of the algorithm to identify correctly the presence or absence of confidence intervals, numerical p-values, p thresholds, and significance language, we estimated a pooled sensitivity and pooled specificity across these four characteristics.

We estimated time trends using weighted nonparametric local regression smoothing (LOESS) [11, 12]. For the LOESS, we calculated 95% confidence intervals using the score method [13] and derived inverse variance weights for additional LOESS weighting. All statistical analyses were done with SAS 9.4 (SAS Institute, Cary, North Carolina).

## Results

**Fig. 1:**
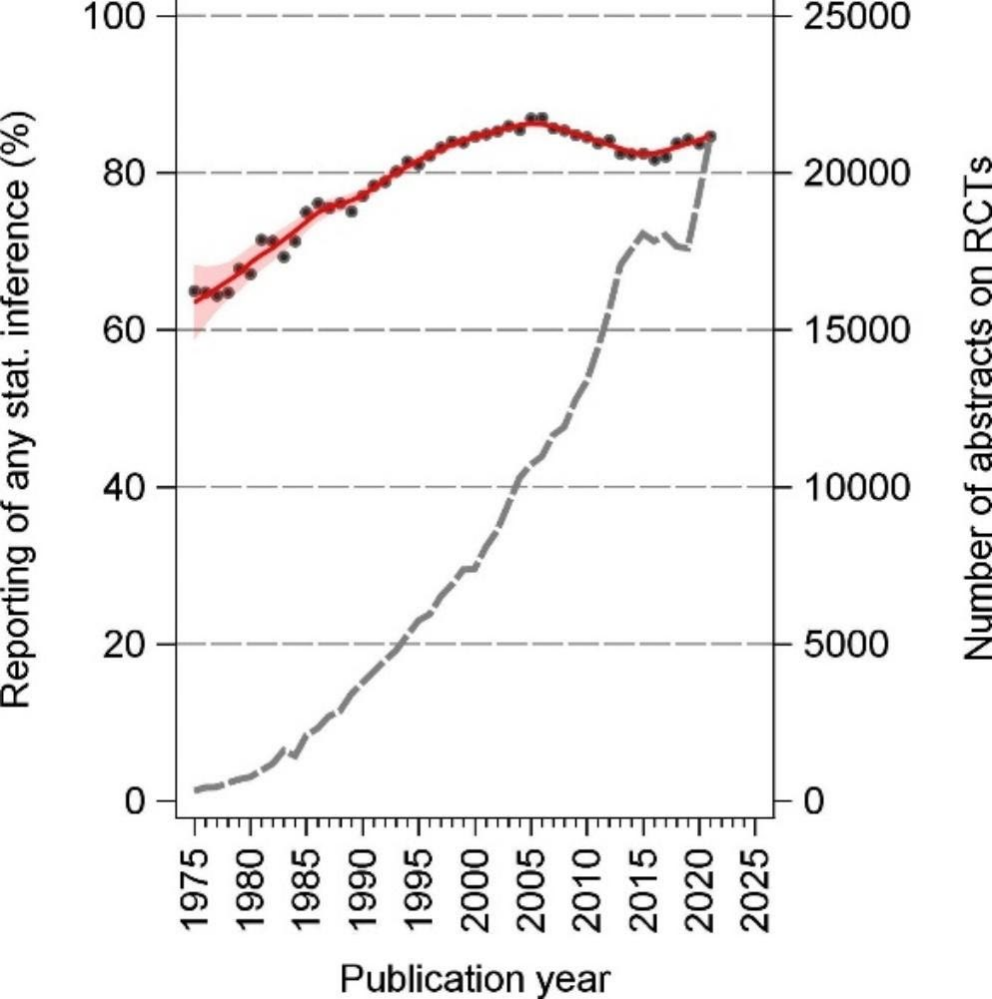
Annual number of abstracts of randomized controlled trials and percentage of abstracts containing statistical inference, 1975–2021 red graph: estimated proportion of abstracts on RCTs that include statistical inference, by year (LOESS with smooth = 0.2) with 95% confidence interval bands; black dots: observed proportion, by year; dashed line: total number of abstracts of RCTs per year

For the period 1975 through 2021, a total of 385 867 abstracts on RCTs were extracted, of which 320 676 (83%) contained some statistical inference. The number of annual abstracts on RCTs increased about 67-fold from 1975 to 2021, with 314 abstracts in 1975 increasing to 21,112 abstracts in 2021. From 2016 to 2019, there was a lull in the increase of abstracts on RCTs. The percentage of abstracts that included any statistical inference increased from 65% to 1975 to a peak of 87% in 2006, and then decreased slightly to 85% in 2021 (Fig. 1).

**Fig. 2:**
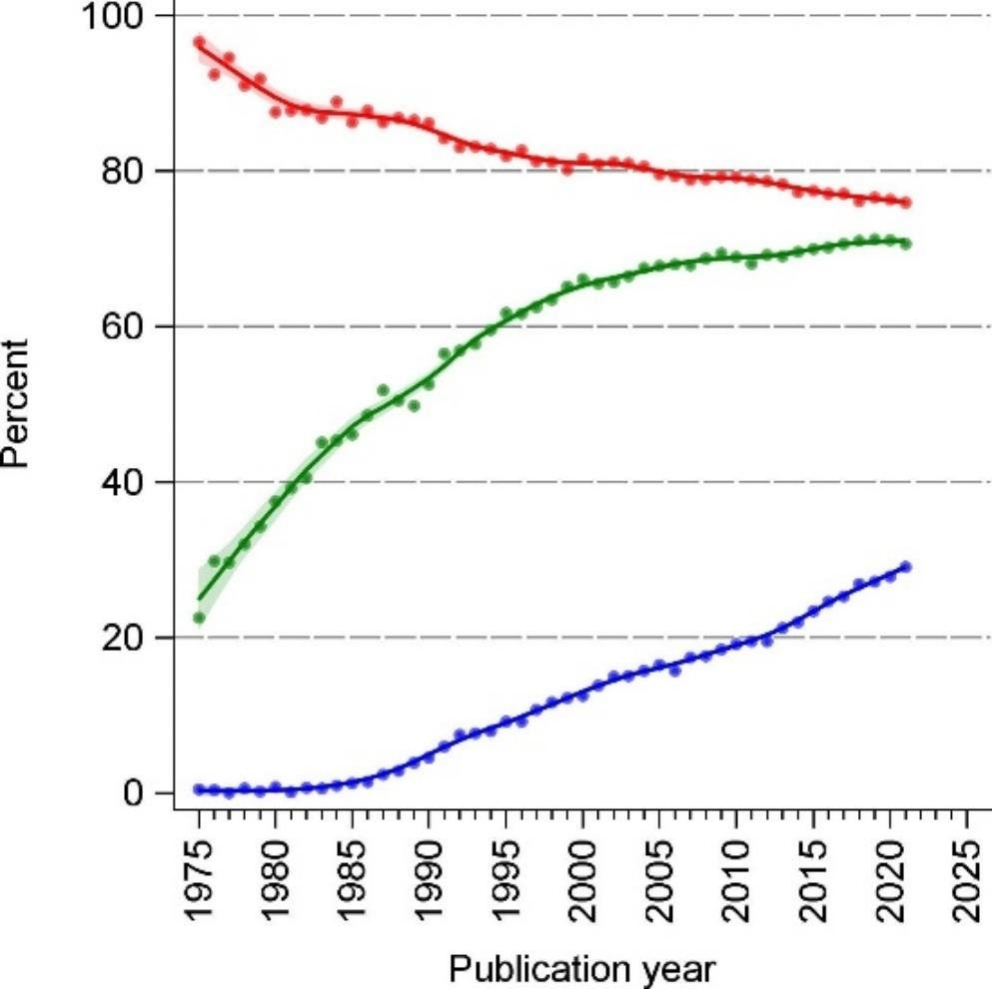
Estimated time trends, 1975–2021, of the statistical reporting style in abstracts on randomized controlled trials (non-hierarchical) **Blue** indicates reporting of confidence intervals; **green** indicates reporting of numerical p-values or p thresholds, (e.g. p < 0.01); **red** indicates reporting of significance language. All trend lines are LOESS smoothed with inverse-variance weighting

Overall and within each calendar time period, significance language was the most common mode of statistical inference. Even in the most recent period, 2015–2021, significance language was used in 77% of all abstracts. The second most common type of reporting statistical inference was the reporting of numerical p-values or p-thresholds, which during 2015–2021 was used in 71% of abstracts. The third mode, overall and in each calendar period, was the reporting of confidence intervals, which during 2015–2021 was present in 26% of abstracts. Among abstracts that included some statistical inference, reporting of significance language decreased over the years, while the reporting of p-values and confidence intervals increased. Starting around 2000, the proportion of abstracts reporting p-values increased much more slowly. Since the mid 1980s, there has been a steady increase in the proportion of abstracts reporting confidence intervals (Table 1; Fig. 2).

**Table 1:** Prevalence of reporting of statistical inference in abstracts related to randomized controlled trials of the publication years 1975–2021 (non-hierarchical)

Period	Total (n)	Any statistical inference n (%)		Percentages among abstracts containing statistical inference (%)^1)^		
				Confidence intervals	p-values	Significance language
**Total period 1975–2021**	**385 867**	**320 676**	**(83.1)**	**18.8**	**66.9**	**79.2**
1975–1979	2430	1594	(65.6)	0.3	30.7	92.9
1980–1984	5947	4180	(70.3)	0.7	42.3	87.8
1985–1989	13 338	10 081	(75.6)	2.6	49.6	86.7
1990–1994	22 395	17 771	(79.4)	6.9	57.0	83.7
1995–1999	32 453	26 928	(83.0)	10.7	63.1	81.4
2000–2004	43 771	37 345	(85.3)	14.6	66.3	81.0
2005–2009	57 988	49 827	(85.9)	17.2	68.4	79.2
2010–2014	78 019	65 081	(83.4)	20.4	69.0	78.5
2015–2021	129 526	107 869	(83.3)	26.4	70.7	76.6

**Fig. 3:**
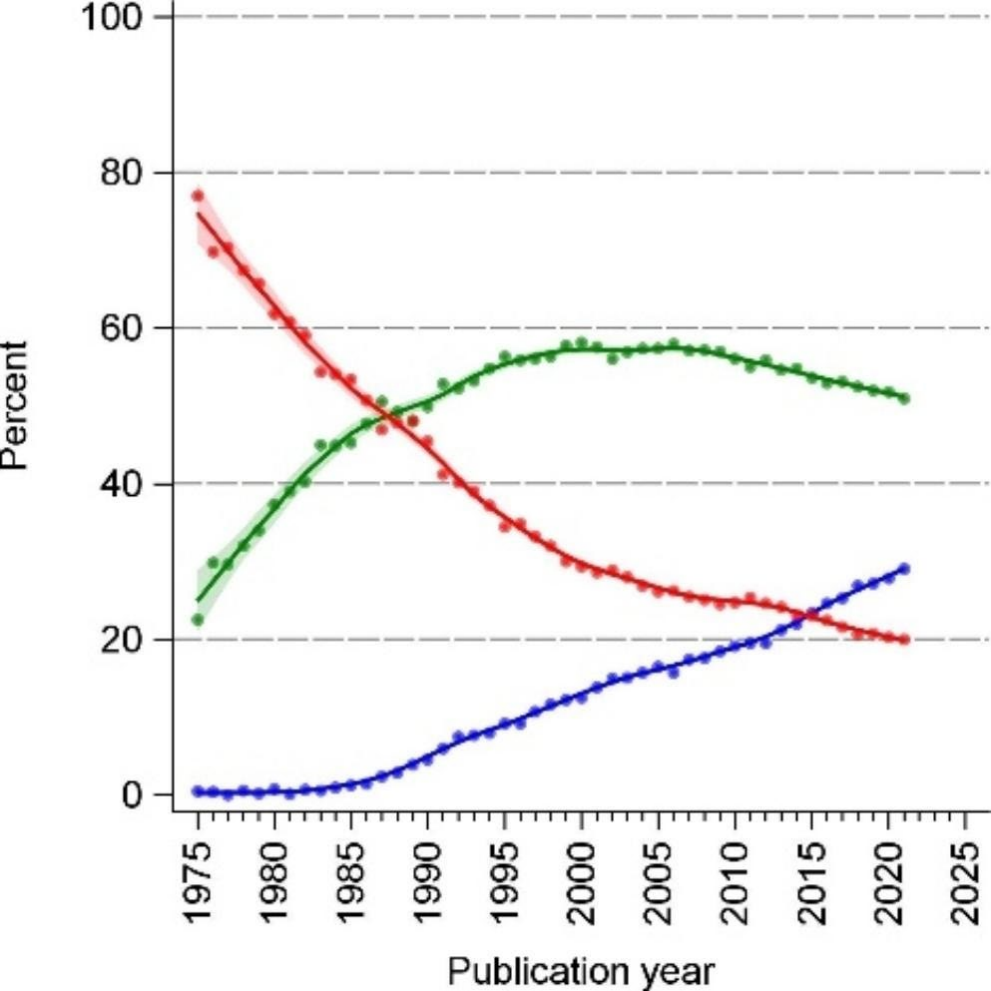
Flexibly estimated time trends 1975–2021 of the statistical reporting style in abstracts on randomized controlled trials (hierarchical) **blue graphs** indicate reporting of confidence intervals; **green graphs** indicate reporting of numerical p-values or p thresholds (e.g. p < 0.01) without confidence intervals; **red graphs** indicate reporting of significance without confidence intervals and p-values; all trend lines are LOESS smoothed with inverse-variance weighting

1) the same abstract may have reported more than one type of statistical inference so that the sum of percentages is above 100%;

When we classified studies using our hierarchy of reporting modes (best: confidence intervals, second best: p-values without confidence intervals, worst: significance language only), we found that during 1975–1990, sole reporting of significance language was predominant. Since 1990, reporting p-values without confidence intervals has been the most common reporting style. The reporting of confidence intervals increased from 0.5% to 1975 to 29% in 2021 (Table 2; Fig. 3).

**Table 2:** Prevalence of reporting of statistical inference in abstracts related to randomized controlled trials of the publication years 1975–2021 (hierarchical)

Period	Total (n)	Any statistical inference n (%)		Percentages among abstracts containing statistical inference (%)		
				Confidence intervals	p-values without confidence intervals	Significance language only
**Total period 1975–2021**	**385 867**	**320 676**	**(83.1)**	**18.8**	**54.3**	**26.9**
1975–1979	2430	1594	(65.6)	0.3	30.6	69.1
1980–1984	5947	4180	(70.3)	0.7	42.1	57.3
1985–1989	13 338	10 081	(75.6)	2.6	48.3	49.1
1990–1994	22 395	17 771	(79.4)	6.9	52.8	40.3
1995–1999	32 453	26 928	(83.0)	10.7	56.5	32.7
2000–2004	43 771	37 345	(85.3)	14.6	57.2	28.3
2005–2009	57 988	49 827	(85.9)	17.2	57.3	25.5
2010–2014	78 019	65 081	(83.4)	20.4	55.2	24.4
2015–2021	129 526	107 869	(83.3)	26.4	52.4	21.2

The proportion of abstracts containing at least one effect-measure estimate for a binary outcome increased over time (1975: 0.2%, 2021: 9.6%). The two most common effect-measure estimates were for hazard ratios and odds ratios. While the reporting of hazard ratio estimates increased steadily, the reporting of odds ratio estimates was already common in the early years after 1975. Reporting of risk ratios estimates has decreased over time. Reporting of both absolute and relative effect-measure estimates as recommended by CONSORT in 2010, occurred infrequently throughout the study period. The proportion of papers with both types of measures was 2.7% during 2015–2021. NNT and NNH are reported in less than 5% of abstracts with binary endpoints (Table 3).

**Table 3:** Prevalence of reporting of various effect-measure estimates in abstracts related to randomized controlled trials of the publication years 1975–2021

Period	Total (n)	Any effect-measure estimate n (%)		Percentages among abstracts containing effect-measure estimates (%)^1)^								
				Hazard Ratio	Rate Ratio	Risk Ratio	Odds Ratio	Rate Diff.	Risk Diff.	NNT	NNH	Rel. & abs.^2)^
**Total period 1975–2021**	**385 867**	**27 144**	**7.0**	**36.0**	**4.4**	**25.6**	**33.6**	**0.6**	**4.3**	**4.1**	**0.2**	**2.0**
1975–1979	2430	5	0.2									
1980–1984	5947	18	0.3									
1985–1989	13 338	88	0.7	4.6	5.7	59.1	28.4	3.4	0.0	0.0	0.0	0.0
1990–1994	22 395	452	2.0	8.9	2.0	57.1	33.2	1.1	1.3	0.0	0.0	1.1
1995–1999	32 453	1243	3.8	12.8	2.1	52.6	33.7	0.2	1.2	0.9	0.0	0.7
2000–2004	43 771	2542	5.8	21.5	2.3	39.0	35.5	0.4	3.2	4.7	0.2	1.7
2005–2009	57 988	4169	7.2	35.0	3.3	29.5	31.9	0.6	3.3	4.9	0.2	1.7
2010–2014	78 019	6227	8.0	40.0	5.0	21.2	33.7	0.5	3.2	4.6	0.3	1.3
2015–2021	129 526	12 400	9.6	41.0	5.3	19.5	33.8	0.8	5.8	3.9	0.3	2.7

(1) the same abstract may have reported more than one type of effect-measure estimate so that the sum of percentages is above 100%; (2) simultaneous reporting of relative and absolute effect-measures estimates

## Discussion

Our analysis of 385 867 abstracts on RCTs from 1975 to 2021 shows a steep increase in the number of abstracts on RCTs each year. The proportion of abstracts reporting statistical inference exclusively declaring that there is a “significant” difference between two groups has gradually and modestly decreased in favor of reporting numerical p-values, and p-thresholds along with confidence intervals. Nonetheless, confidence intervals are reported markedly less frequently than p-values. Within abstracts reporting effect-measure estimates on binary outcomes, reporting of hazard ratio estimates shows the greatest increase over time. The reporting of NNT and NNH estimates has not gained traction. The reporting of odds ratio estimates is found in about one third of all abstracts on RCTs that report effect-measure estimates on dichotomous outcomes.

A text mining project of 1.6 million PubMed abstracts and 385 000 PMC full texts of biomedical research articles published between 1990 and 2015 found that reporting of p-values (numerical or threshold, e.g. p < 0.05 or p ≤ 0.05) in abstracts has increased from 7.3% to 1990 to 15.6% in 2014 [14]. Within the group of abstracts reporting a p-value (numerical or threshold, e.g. p < 0.05 or p ≤ 0.05), the proportion of abstracts reporting a p-threshold decreased over time in favor of reporting numerical p-values [14]. Previous systematic reviews on the reporting style of statistical inference in selected major medical and epidemiological journals [7], psychiatry journals [8], cardiology journals [10], clinical pharmacology journals [9], and cancer journals [15] showed that the percentage of confidence intervals in abstracts containing statistical inference has increased, even if reporting of p-values dominates. Even in 2021, a confidence interval is reported for effect-measure estimates for binary outcomes in a minority of RCT abstracts. Thus, for the majority of RCT abstracts, readers have no immediate way to assess statistical uncertainty of effect-measure estimates. Failure to report CIs may reflect a lack of appreciation by authors, reviewers or editors for the information that the interval estimates convey.

In a review in 2008 of 193 publications in five major general medical journals of RCTs that used binary primary outcomes, OR estimates were reported in 12%. A total of 14% reported OR estimates for other outcomes or for subgroup analyses [16]. Another paper analyzed 580 publications of RCTs in the New England Journal of Medicine from 2004 to 2014. In a subset of publications where RR estimates could be calculated from reported OR estimates, the OR estimate was found to overestimate the RR estimate in 62% of cases. The overestimation was > 50% in 28 RCTs and > 100% in 13 RCTs [17]. Rombach et al., in an analysis of 200 publications on RCTs, found that only 55% of publications reported an effect-measure estimate at all [18]. One of the earliest publications to point out that the OR deviates from the RR when the risk of the outcome is substantial in at least one of the study arms was from Cornfield [19]. The assessment of how rare a condition should be depends on the tolerance regarding the approximation error. For example, if one wants the error to be no greater than 10% for the RR, the risk of the outcome should be no greater than 10% in each study arm [20]. In 2011, Knol et al. reported extreme cases of abstracts in which the OR estimate deviated considerably from the RR estimate in a RCT [16]. We found one example in which the estimated risk of the outcome was 95% in one study arm and 68% in the other [21], for a risk difference estimate of 27%. The authors did not report risk difference, however. Rather, they reported an OR estimate of 9.3. Had they reported RR instead, it would have been 0.95/0.68 = 1.4. The deviation of the OR from the RR is influenced not only by the rarity of the outcome in all treatment levels, but also by the difference in risks between the study arms. The difference between OR and RR may remain small even with high risks in the study arms as long as the risks are similar (e.g., risk in one study arm 72%, in the other study arm 70%, OR = 1.10, RR = 1.03, risk difference + 2% points). ORs in abstracts of RCTs may come from embedded case-control studies, from cross-sectional analyses (prevalence OR), or from longitudinal analyses of RCT data and may therefore have different interpretations.

Interestingly, Kolaski et al. (2023) cite work showing that authors often label their study design incompletely or inaccurately, resulting in incorrect indexing of papers in PubMed and other literature databases [22]. We used “publication types” keyworded by the National Library of Medicine in our search strategy of publications on RCTs. We also combined that with text word searches to improve recall (albeit at the expense of search precision). This may explain why our review also included case-control studies embedded in RCTs or secondary analyses for prognostic and treatment prediction models based on RCT data in some cases.

Whereas absolute effect measures gauge clinical importance and public health importance in general, relative effect measures obscure it [23, 24]. In their code of practice for the pharmaceutical industry, the Association of the British Pharmaceutical Industry (ABPI) states: “Referring only to relative risk, especially with regard to risk reduction, can make a medicine appear more effective than it actually is. In order to assess the clinical impact of an outcome, the reader also needs to know the absolute risk involved. In that regard relative risk should never be referred to without also referring to the absolute risk. Absolute risk can be referred to in isolation” [23]. Nevertheless, it remains a rarity for studies to report absolute effect measure estimates (i.e., difference measures) for binary outcomes, or to report both absolute and relative effect-measure estimates as recommended by CONSORT in 2010. In a review of 359 full papers published in general medical journals in 1989, 1992, 1995, and 1998, that reported results from RCTs and mentioned a statistically significant treatment effect, absolute effect measures were reported in 5.0% and NNT was reported in 2.2% [25].

In 2016, the American Statistical Association explicitly stated, “The widespread use of ‘statistical significance’ (generally interpreted as ‘p ≤ 0.05’) as a license for making a claim of a scientific finding (or implied truth) leads to considerable distortion of the scientific process.” [26]. More recently, the ASA provided an even stronger statement that “it is time to stop using the term ‘statistically significant’ entirely. Nor should variants such as ‘significantly different,’ ‘p < 0.05,’ and ‘nonsignificant’ survive, whether expressed in words, by asterisks in a table, or in some other way. Whether it was ever useful, a declaration of ‘statistical significance’ has today become meaningless” [27]. It remains unclear why confidence intervals around effect-measure estimates are so rarely reported in abstracts of RCTs, although the CONSORT guideline has long called for this and the ASA explicitly discourages making decisions based on statistical significance.

Our study involved the complete review of all RCT abstracts from 1975 to 2021 (385 867 abstracts) and the use of a validated text-mining algorithm that automatically detected the reporting of statistical inferences, the statistical reporting style of outcomes, and effect-measure estimates for binary disease outcomes. Nonetheless, our analysis has also several important limitations. First, we only studied abstracts of RCTs, which may not capture the reporting style of the full text. For example, based on 300 abstracts from the three leading clinical pharmacology journals from 2012 to 2016, 50% of the abstracts contained statistical inference, whereas in the full text of the same publications, 88% contained statistical inference. The proportion of reporting confidence intervals in abstracts that contained statistical inference was also lower (45%) than in the full texts of the same publications (58%) [9]. Our rationale to focus on abstracts was that (1) the reporting style in abstracts reflects the results that authors consider most noteworthy, (2) the abstract is often the only part of a publication that is read, and (3) although proper presentation and interpretation of study results is relevant throughout the manuscript, it is especially relevant in abstracts [28]. Second, our PubMed algorithm identified not only RCT abstracts that contained comparative analyses on the primary endpoint, but also post-hoc analyses of RCT data (e.g. prognostic prediction models, nested case-control studies in RCTs), and also protocols on RCTs. For this reason, we limited our analyses of the reporting style of statistical inference to abstracts that contained some statistical inference. Similarly, with regard to the reporting of effect-measure estimates for binary outcomes, we restricted our analysis to abstracts in which an effect-measure estimate for binary outcomes was present at all. Third, our text-mining algorithms were not perfect. We therefore used time-stratified random samples of abstracts to validate the algorithms. For example, the error rate of the categorization of statistical reporting style among abstracts containing statistical inference was 2.5%. The text-mining algorithm regarding the detection of effect-measure estimates of interest worked nearly perfectly.

### Electronic Supplementary Material

Below is the link to the electronic supplementary material.

Supplementary Material 1
