# Supplementary material for: Statistical inference and effect measures in abstracts of randomized controlled trials, 1975–2021. A systematic review
Source: Eur J Epidemiol. 2023 Sep 16;38(10):1035–42. doi: 10.1007/s10654-023-01047-8 (PMC10570208; doi:10.1007/s10654-023-01047-8)
Supplement: Supplementary file 1 — Supplementary Material 1 [file 10654_2023_1047_MOESM1_ESM.docx]

**Supplement**

**PubMed search algorithm**((("randomized controlled trial"[Publication Type] AND "hasabstract"[All Fields] AND "English"[Language] AND 1975/01/01:2021/12/31[Date - Publication] AND ("randomized"[Title/Abstract] OR "RANDOMIZED"[Title/Abstract] OR "randomised"[Title/Abstract] OR "randomization"[Title/Abstract] OR "randomisation"[Title/Abstract] OR "randomizing"[Title/Abstract] OR "randomising"[Title/Abstract] OR "randomly"[Title/Abstract])) NOT "clinical trial, phase i"[Publication Type]) NOT "clinical trial, phase ii"[Publication Type])

**Suppl. Table 1 Types of errors of the algorithm to identify the four characteristics related to statistical inference (18 errors among 180 abstracts)**

| Error no. | PMID | Comment |
| --- | --- | --- |
| ***11 False-positive errors of the algorithm that believed it is statistical significance*** | | |
| 1 | 7383488 | “significantly affects mothering” |
| 2 | 6409599 | “no significant carryover effect were observed” |
| 3 | 3329078 | “clinically significant” |
| 4 | 3342553 | “no significant adverse drug reactions” |
| 5 | 2192700 | “no clinically significant” |
| 6 | 1704153 | “Control of the effusion significantly reduces morbidity” |
| 7 | 8092591 | “There were no significant adverse effects in either group” |
| 8 | 8963892 | “presence of significant structural heart disease” |
| 9 | 12469979 | “No significant accumulation occurs after multiple doses” |
| 10 | 15451546 | “has no clinical significance” |
| 11 | 17544889 | “There were no significant adverse events related to CVCM application” |
| ***7 Errors related to other problems*** | | |
| 12 | 1247310 | “Pless than 0.005” was not detected as p threshold reporting [the missing empty space between P and less was a problem here] |
| 13 | 362900 | “significant at the .05 level” was not detected as p threshold reporting |
| 14 | 1920342 | “DBP less than” was incorrectly interpreted as “P less than” |
| 15 | 8963892 | “P = NS” was incorrectly interpreted as precise p-value reporting |
| 16 | 11728069 | “95 CI” was not discovered as confidence interval reporting as the percentage symbol was missing [authors used uncommon abbreviation] |
| 17 | 12612386 | “hazard ratio was 0.79 (0.52 1.21)” was not discovered as confidence interval reporting [authors did not indicate what they report in parentheses] |

**Suppl. Table 2 Estimated sensitivity and specificity of the algorithm to identify the four characteristics related to statistical inference among 180 abstracts**

| **Reporting style** | **Sensitivity** | **%** | **Specificity** | **%** |
| --- | --- | --- | --- | --- |
| Confidence interval reporting | 16/19 | 84 | 161/161 | 100 |
| Numerical p-value reporting | 58/58 | 100 | 121/122 | 99 |
| P threshold reporting | 71/73 | 97 | 106/107 | 99 |
| Significance terminology | 134/134 | 100 | 35/46 | 75 |
|  |  |  |  |  |
| Across all four items | 279/284 | 98 | 423/436 | 97 |
